# Supplementary material for: Multi-omics analyses reveal that the gut microbiome and its metabolites promote milk fat synthesis in Zhongdian yak cows
Source: PeerJ. 2022 Dec 2;10:e14444. doi: 10.7717/peerj.14444 (PMC9744170; doi:10.7717/peerj.14444)
Supplement: Supplemental Information 13 [file peerj-10-14444-s013.zip › Web_Report/HTML/meta_kegg_anno_categroy.xls.html]

meta\_kegg\_anno\_categroy.xls


## meta\_kegg\_anno\_categroy.xls

| KO\_Pathway\_Level1 | KO\_Pathway\_Level2 | Meta\_Num | Metabolites |
| --- | --- | --- | --- |
| Aging | 04211 Longevity regulating pathway | 1 | pos\_2601 |
| Aging | 04212 Longevity regulating pathway - worm | 2 | pos\_2601 pos\_499 |
| Aging | 04213 Longevity regulating pathway - multiple species | 2 | pos\_2601 pos\_499 |
| Amino acid metabolism | 00220 Arginine biosynthesis | 23 | pos\_5 pos\_1675 pos\_1048 pos\_2 pos\_862 pos\_39 pos\_2805 pos\_614 pos\_1898 pos\_2274 pos\_835 pos\_766 pos\_709 pos\_1044 pos\_1315 pos\_671 pos\_1674 pos\_556 pos\_1717 pos\_2105 pos\_2599 pos\_529 pos\_18 |
| Amino acid metabolism | 00250 Alanine, aspartate and glutamate metabolism | 2 | pos\_5 pos\_614 |
| Amino acid metabolism | 00260 Glycine, serine and threonine metabolism | 7 | pos\_2 pos\_2105 pos\_5 pos\_39 pos\_614 pos\_1898 pos\_1048 |
| Amino acid metabolism | 00270 Cysteine and methionine metabolism | 10 | pos\_2 pos\_2105 pos\_5 pos\_39 pos\_1675 pos\_18 pos\_671 pos\_614 pos\_1898 pos\_1048 |
| Amino acid metabolism | 00280 Valine, leucine and isoleucine degradation | 11 | pos\_2 pos\_2105 pos\_5 pos\_39 pos\_1044 pos\_1675 pos\_18 pos\_671 pos\_614 pos\_1898 pos\_1048 |
| Amino acid metabolism | 00290 Valine, leucine and isoleucine biosynthesis | 13 | pos\_2 pos\_1717 pos\_2105 pos\_5 pos\_39 pos\_1044 pos\_1315 pos\_1675 pos\_18 pos\_671 pos\_614 pos\_1898 pos\_1048 |
| Amino acid metabolism | 00300 Lysine biosynthesis | 15 | pos\_2 pos\_1717 pos\_862 pos\_2105 pos\_5 pos\_39 pos\_2805 pos\_1044 pos\_1315 pos\_1675 pos\_18 pos\_671 pos\_614 pos\_1898 pos\_1048 |
| Amino acid metabolism | 00310 Lysine degradation | 20 | pos\_5 pos\_1675 pos\_1048 pos\_2 pos\_862 pos\_39 pos\_2805 pos\_614 pos\_1898 pos\_835 pos\_709 pos\_1044 pos\_1315 pos\_671 pos\_1674 pos\_556 pos\_1717 pos\_2105 pos\_529 pos\_18 |
| Amino acid metabolism | 00330 Arginine and proline metabolism | 28 | pos\_5 pos\_913 pos\_1675 pos\_1048 pos\_2 pos\_862 pos\_39 pos\_2805 pos\_614 pos\_1898 pos\_2274 pos\_835 pos\_766 pos\_1027 pos\_709 pos\_1044 pos\_4 pos\_1315 pos\_671 pos\_1674 pos\_650 pos\_556 pos\_1717 pos\_980 pos\_2105 pos\_2599 pos\_529 pos\_18 |
| Amino acid metabolism | 00340 Histidine metabolism | 38 | pos\_816 pos\_1267 pos\_5 pos\_913 pos\_1675 pos\_2730 pos\_1899 pos\_1048 pos\_2 pos\_862 pos\_39 pos\_2805 pos\_975 pos\_1264 pos\_2541 pos\_614 pos\_1898 pos\_2274 pos\_835 pos\_766 pos\_1027 pos\_709 pos\_2734 pos\_1044 pos\_4 pos\_1315 pos\_671 pos\_1674 pos\_650 pos\_556 pos\_1717 pos\_980 pos\_2105 pos\_784 pos\_2599 pos\_529 pos\_18 pos\_1275 |
| Amino acid metabolism | 00350 Tyrosine metabolism | 42 | pos\_816 pos\_1267 pos\_5 pos\_913 pos\_1675 pos\_2730 pos\_1899 pos\_1048 pos\_2 pos\_862 pos\_2687 pos\_16 pos\_39 pos\_2805 pos\_975 pos\_1817 pos\_1264 pos\_2541 pos\_614 pos\_1898 pos\_2274 pos\_835 pos\_766 pos\_1027 pos\_709 pos\_2734 pos\_1044 pos\_4 pos\_1315 pos\_671 pos\_1674 pos\_650 pos\_556 pos\_36 pos\_1717 pos\_980 pos\_2105 pos\_784 pos\_2599 pos\_529 pos\_18 pos\_1275 |
| Amino acid metabolism | 00360 Phenylalanine metabolism | 48 | pos\_2572 pos\_816 pos\_1267 pos\_5 pos\_913 pos\_1675 pos\_2730 pos\_1899 pos\_1048 pos\_2 pos\_862 pos\_2687 pos\_16 pos\_39 pos\_2805 pos\_975 pos\_1817 pos\_1264 pos\_2541 pos\_2048 pos\_614 pos\_1898 pos\_2274 pos\_835 pos\_766 pos\_1027 pos\_709 pos\_2734 pos\_1044 pos\_4 pos\_1315 pos\_671 pos\_1674 pos\_650 pos\_556 pos\_36 pos\_1717 pos\_980 pos\_2105 pos\_784 pos\_2599 pos\_529 pos\_2788 pos\_2279 pos\_18 pos\_1275 pos\_1955 pos\_112 |
| Amino acid metabolism | 00380 Tryptophan metabolism | 51 | pos\_2572 pos\_816 pos\_1267 pos\_5 pos\_913 pos\_1675 pos\_2730 pos\_1899 pos\_1048 pos\_2 pos\_862 pos\_2687 pos\_16 pos\_39 pos\_2805 pos\_975 pos\_1817 pos\_1264 pos\_2541 pos\_2048 pos\_2413 pos\_614 pos\_1898 pos\_2274 pos\_835 pos\_766 pos\_1027 pos\_709 pos\_2734 pos\_1044 pos\_4 pos\_1541 pos\_1315 pos\_671 pos\_1674 pos\_650 pos\_556 pos\_36 pos\_1717 pos\_980 pos\_2105 pos\_784 pos\_2599 pos\_529 pos\_2788 pos\_2279 pos\_2548 pos\_18 pos\_1275 pos\_1955 pos\_112 |
| Amino acid metabolism | 00400 Phenylalanine, tyrosine and tryptophan biosynthesis | 53 | pos\_2572 pos\_816 pos\_1267 pos\_5 pos\_913 pos\_1675 pos\_2730 pos\_2768 pos\_1899 pos\_1048 pos\_2 pos\_862 pos\_2687 pos\_16 pos\_39 pos\_2805 pos\_975 pos\_1817 pos\_1264 pos\_2541 pos\_2048 pos\_2413 pos\_614 pos\_1898 pos\_2274 pos\_835 pos\_766 pos\_1027 pos\_709 pos\_2734 pos\_431 pos\_1044 pos\_4 pos\_1541 pos\_1315 pos\_671 pos\_1674 pos\_650 pos\_556 pos\_36 pos\_1717 pos\_980 pos\_2105 pos\_784 pos\_2599 pos\_529 pos\_2788 pos\_2279 pos\_2548 pos\_18 pos\_1275 pos\_1955 pos\_112 |
| Biosynthesis of other secondary metabolites | 00232 Caffeine metabolism | 2 | pos\_597 pos\_1569 |
| Biosynthesis of other secondary metabolites | 00254 Aflatoxin biosynthesis | 5 | pos\_766 pos\_2575 pos\_1569 pos\_597 pos\_1048 |
| Biosynthesis of other secondary metabolites | 00261 Monobactam biosynthesis | 4 | pos\_597 pos\_1569 pos\_766 pos\_1048 |
| Biosynthesis of other secondary metabolites | 00311 Penicillin and cephalosporin biosynthesis | 2 | pos\_597 pos\_1569 |
| Biosynthesis of other secondary metabolites | 00331 Clavulanic acid biosynthesis | 4 | pos\_597 pos\_1569 pos\_766 pos\_1048 |
| Biosynthesis of other secondary metabolites | 00332 Carbapenem biosynthesis | 2 | pos\_597 pos\_1569 |
| Biosynthesis of other secondary metabolites | 00333 Prodigiosin biosynthesis | 5 | pos\_766 pos\_2575 pos\_1569 pos\_597 pos\_1048 |
| Biosynthesis of other secondary metabolites | 00401 Novobiocin biosynthesis | 5 | pos\_766 pos\_2575 pos\_1569 pos\_597 pos\_1048 |
| Biosynthesis of other secondary metabolites | 00402 Benzoxazinoid biosynthesis | 2 | pos\_597 pos\_1569 |
| Biosynthesis of other secondary metabolites | 00404 Staurosporine biosynthesis | 5 | pos\_766 pos\_2575 pos\_1569 pos\_597 pos\_1048 |
| Biosynthesis of other secondary metabolites | 00405 Phenazine biosynthesis | 5 | pos\_766 pos\_2575 pos\_1569 pos\_597 pos\_1048 |
| Biosynthesis of other secondary metabolites | 00521 Streptomycin biosynthesis | 4 | pos\_597 pos\_1569 pos\_766 pos\_1048 |
| Biosynthesis of other secondary metabolites | 00524 Neomycin, kanamycin and gentamicin biosynthesis | 5 | pos\_766 pos\_2575 pos\_1569 pos\_597 pos\_1048 |
| Biosynthesis of other secondary metabolites | 00525 Acarbose and validamycin biosynthesis | 5 | pos\_766 pos\_2575 pos\_1569 pos\_597 pos\_1048 |
| Biosynthesis of other secondary metabolites | 00965 Betalain biosynthesis | 2 | pos\_597 pos\_1569 |
| Biosynthesis of other secondary metabolites | 00966 Glucosinolate biosynthesis | 2 | pos\_597 pos\_1569 |
| Biosynthesis of other secondary metabolites | 00997 Biosynthesis of various secondary metabolites - part 3 | 5 | pos\_766 pos\_2575 pos\_1569 pos\_597 pos\_1048 |
| Biosynthesis of other secondary metabolites | 00998 Biosynthesis of various secondary metabolites - part 2 | 5 | pos\_766 pos\_2575 pos\_1569 pos\_597 pos\_1048 |
| Biosynthesis of other secondary metabolites | 00999 Biosynthesis of various secondary metabolites - part 1 | 5 | pos\_766 pos\_2575 pos\_1569 pos\_597 pos\_1048 |
| Cancer: overview | 05200 Pathways in cancer | 7 | pos\_1442 pos\_662 pos\_1982 pos\_1165 pos\_1169 pos\_9 pos\_614 |
| Cancer: overview | 05202 Transcriptional misregulation in cancer | 7 | pos\_1442 pos\_662 pos\_1982 pos\_1165 pos\_1169 pos\_9 pos\_614 |
| Cancer: overview | 05203 Viral carcinogenesis | 7 | pos\_1442 pos\_662 pos\_1982 pos\_1165 pos\_1169 pos\_9 pos\_614 |
| Cancer: overview | 05204 Chemical carcinogenesis - DNA adducts | 7 | pos\_1442 pos\_662 pos\_1982 pos\_1165 pos\_1169 pos\_9 pos\_614 |
| Cancer: overview | 05205 Proteoglycans in cancer | 7 | pos\_1442 pos\_662 pos\_1982 pos\_1165 pos\_1169 pos\_9 pos\_614 |
| Cancer: overview | 05206 MicroRNAs in cancer | 7 | pos\_1442 pos\_662 pos\_1982 pos\_1165 pos\_1169 pos\_9 pos\_614 |
| Cancer: overview | 05207 Chemical carcinogenesis - receptor activation | 7 | pos\_1442 pos\_662 pos\_1982 pos\_1165 pos\_1169 pos\_9 pos\_614 |
| Cancer: overview | 05230 Central carbon metabolism in cancer | 14 | pos\_766 pos\_2105 pos\_1267 pos\_1442 pos\_5 pos\_662 pos\_1982 pos\_1044 pos\_4 pos\_1165 pos\_1169 pos\_9 pos\_671 pos\_614 |
| Cancer: overview | 05231 Choline metabolism in cancer | 22 | pos\_1267 pos\_5 pos\_1169 pos\_39 pos\_1982 pos\_1833 pos\_2769 pos\_1806 pos\_614 pos\_766 pos\_1044 pos\_1430 pos\_4 pos\_9 pos\_671 pos\_1018 pos\_2263 pos\_2105 pos\_1442 pos\_662 pos\_1165 pos\_1619 |
| Cancer: overview | 05235 PD-L1 expression and PD-1 checkpoint pathway in cancer | 22 | pos\_1267 pos\_5 pos\_1169 pos\_39 pos\_1982 pos\_1833 pos\_2769 pos\_1806 pos\_614 pos\_766 pos\_1044 pos\_1430 pos\_4 pos\_9 pos\_671 pos\_1018 pos\_2263 pos\_2105 pos\_1442 pos\_662 pos\_1165 pos\_1619 |
| Cancer: specific types | 05211 Renal cell carcinoma | 4 | pos\_1442 pos\_662 pos\_9 pos\_614 |
| Cancer: specific types | 05213 Endometrial cancer | 6 | pos\_1442 pos\_662 pos\_1165 pos\_9 pos\_1169 pos\_614 |
| Cancer: specific types | 05214 Glioma | 3 | pos\_1442 pos\_662 pos\_9 |
| Cancer: specific types | 05215 Prostate cancer | 6 | pos\_1442 pos\_662 pos\_1165 pos\_9 pos\_1169 pos\_614 |
| Cancer: specific types | 05216 Thyroid cancer | 3 | pos\_1442 pos\_662 pos\_9 |
| Cancer: specific types | 05217 Basal cell carcinoma | 3 | pos\_1442 pos\_662 pos\_9 |
| Cancer: specific types | 05218 Melanoma | 3 | pos\_1442 pos\_662 pos\_9 |
| Cancer: specific types | 05219 Bladder cancer | 4 | pos\_1442 pos\_662 pos\_9 pos\_614 |
| Cancer: specific types | 05220 Chronic myeloid leukemia | 3 | pos\_1442 pos\_662 pos\_9 |
| Cancer: specific types | 05221 Acute myeloid leukemia | 3 | pos\_1442 pos\_662 pos\_9 |
| Cancer: specific types | 05222 Small cell lung cancer | 6 | pos\_1442 pos\_662 pos\_1165 pos\_9 pos\_1169 pos\_614 |
| Cancer: specific types | 05223 Non-small cell lung cancer | 6 | pos\_1442 pos\_662 pos\_1165 pos\_9 pos\_1169 pos\_614 |
| Cancer: specific types | 05224 Breast cancer | 6 | pos\_1442 pos\_662 pos\_1165 pos\_9 pos\_1169 pos\_614 |
| Cancer: specific types | 05225 Hepatocellular carcinoma | 1 | pos\_1442 |
| Cancer: specific types | 05226 Gastric cancer | 3 | pos\_1442 pos\_662 pos\_9 |
| Carbohydrate metabolism | 00020 Citrate cycle (TCA cycle) | 2 | pos\_5 pos\_614 |
| Carbohydrate metabolism | 00030 Pentose phosphate pathway | 3 | pos\_5 pos\_2731 pos\_614 |
| Carbohydrate metabolism | 00040 Pentose and glucuronate interconversions | 6 | pos\_5 pos\_752 pos\_2731 pos\_52 pos\_1129 pos\_614 |
| Carbohydrate metabolism | 00051 Fructose and mannose metabolism | 6 | pos\_5 pos\_752 pos\_2731 pos\_52 pos\_1129 pos\_614 |
| Carbohydrate metabolism | 00052 Galactose metabolism | 8 | pos\_5 pos\_2785 pos\_752 pos\_2731 pos\_52 pos\_1129 pos\_1087 pos\_614 |
| Carbohydrate metabolism | 00053 Ascorbate and aldarate metabolism | 8 | pos\_5 pos\_2785 pos\_752 pos\_2731 pos\_52 pos\_1129 pos\_1087 pos\_614 |
| Carbohydrate metabolism | 00500 Starch and sucrose metabolism | 10 | pos\_5 pos\_2785 pos\_2409 pos\_752 pos\_2731 pos\_52 pos\_1129 pos\_1087 pos\_19 pos\_614 |
| Carbohydrate metabolism | 00520 Amino sugar and nucleotide sugar metabolism | 12 | pos\_252 pos\_5 pos\_2785 pos\_2409 pos\_477 pos\_752 pos\_2731 pos\_52 pos\_1129 pos\_1087 pos\_19 pos\_614 |
| Carbohydrate metabolism | 00562 Inositol phosphate metabolism | 15 | pos\_1717 pos\_2105 pos\_252 pos\_5 pos\_2785 pos\_2409 pos\_477 pos\_752 pos\_2731 pos\_52 pos\_1129 pos\_1087 pos\_19 pos\_614 pos\_717 |
| Carbohydrate metabolism | 00620 Pyruvate metabolism | 13 | pos\_1717 pos\_252 pos\_5 pos\_2785 pos\_2409 pos\_477 pos\_752 pos\_2731 pos\_52 pos\_1129 pos\_1087 pos\_19 pos\_614 |
| Carbohydrate metabolism | 00630 Glyoxylate and dicarboxylate metabolism | 14 | pos\_1717 pos\_2105 pos\_252 pos\_5 pos\_2785 pos\_2409 pos\_477 pos\_752 pos\_2731 pos\_52 pos\_1129 pos\_1087 pos\_19 pos\_614 |
| Carbohydrate metabolism | 00640 Propanoate metabolism | 15 | pos\_1717 pos\_2105 pos\_252 pos\_5 pos\_2785 pos\_2409 pos\_477 pos\_752 pos\_2731 pos\_52 pos\_1129 pos\_1087 pos\_19 pos\_614 pos\_717 |
| Carbohydrate metabolism | 00650 Butanoate metabolism | 15 | pos\_1717 pos\_2105 pos\_252 pos\_5 pos\_2785 pos\_2409 pos\_477 pos\_752 pos\_2731 pos\_52 pos\_1129 pos\_1087 pos\_19 pos\_614 pos\_717 |
| Carbohydrate metabolism | 00660 C5-Branched dibasic acid metabolism | 15 | pos\_1717 pos\_2105 pos\_252 pos\_5 pos\_2785 pos\_2409 pos\_477 pos\_752 pos\_2731 pos\_52 pos\_1129 pos\_1087 pos\_19 pos\_614 pos\_717 |
| Cell growth and death | 04115 p53 signaling pathway | 3 | pos\_1652 pos\_1709 pos\_1714 |
| Cell growth and death | 04216 Ferroptosis | 2 | pos\_1652 pos\_1714 |
| Cell growth and death | 04217 Necroptosis | 3 | pos\_1652 pos\_1709 pos\_1714 |
| Cell growth and death | 04218 Cellular senescence | 3 | pos\_1652 pos\_1709 pos\_1714 |
| Cell motility | 04810 Regulation of actin cytoskeleton | 1 | pos\_879 |
| Cellular community - eukaryotes | 04540 Gap junction | 1 | pos\_1442 |
| Cellular community - eukaryotes | 04550 Signaling pathways regulating pluripotency of stem cells | 1 | pos\_1442 |
| Circulatory system | 04261 Adrenergic signaling in cardiomyocytes | 1 | pos\_1442 |
| Circulatory system | 04270 Vascular smooth muscle contraction | 3 | pos\_1442 pos\_1025 pos\_1714 |
| Development and regeneration | 04361 Axon regeneration | 2 | pos\_1442 pos\_884 |
| Development and regeneration | 04380 Osteoclast differentiation | 2 | pos\_1442 pos\_884 |
| Digestive system | 04970 Salivary secretion | 2 | pos\_1442 pos\_879 |
| Digestive system | 04971 Gastric acid secretion | 3 | pos\_1442 pos\_879 pos\_2730 |
| Digestive system | 04972 Pancreatic secretion | 3 | pos\_1442 pos\_879 pos\_2730 |
| Digestive system | 04973 Carbohydrate digestion and absorption | 14 | pos\_2854 pos\_2135 pos\_10 pos\_1442 pos\_913 pos\_39 pos\_1982 pos\_730 pos\_752 pos\_52 pos\_1129 pos\_3 pos\_2730 pos\_879 |
| Digestive system | 04974 Protein digestion and absorption | 24 | pos\_10 pos\_1267 pos\_913 pos\_52 pos\_3 pos\_2730 pos\_879 pos\_1048 pos\_2854 pos\_2135 pos\_862 pos\_39 pos\_1982 pos\_752 pos\_766 pos\_2062 pos\_730 pos\_1044 pos\_4 pos\_1129 pos\_671 pos\_36 pos\_2105 pos\_1442 |
| Digestive system | 04975 Fat digestion and absorption | 24 | pos\_10 pos\_1267 pos\_913 pos\_52 pos\_3 pos\_2730 pos\_879 pos\_1048 pos\_2854 pos\_2135 pos\_862 pos\_39 pos\_1982 pos\_752 pos\_766 pos\_2062 pos\_730 pos\_1044 pos\_4 pos\_1129 pos\_671 pos\_36 pos\_2105 pos\_1442 |
| Digestive system | 04976 Bile secretion | 14 | pos\_2854 pos\_2135 pos\_10 pos\_1442 pos\_913 pos\_39 pos\_1982 pos\_730 pos\_752 pos\_52 pos\_1129 pos\_3 pos\_2730 pos\_879 |
| Digestive system | 04977 Vitamin digestion and absorption | 30 | pos\_10 pos\_1267 pos\_913 pos\_52 pos\_871 pos\_3 pos\_2601 pos\_2730 pos\_879 pos\_1048 pos\_2854 pos\_2135 pos\_862 pos\_39 pos\_1982 pos\_752 pos\_766 pos\_2062 pos\_730 pos\_1044 pos\_4 pos\_1129 pos\_1844 pos\_671 pos\_57 pos\_220 pos\_36 pos\_2105 pos\_1442 pos\_754 |
| Digestive system | 04978 Mineral absorption | 30 | pos\_10 pos\_1267 pos\_913 pos\_52 pos\_871 pos\_3 pos\_2601 pos\_2730 pos\_879 pos\_1048 pos\_2854 pos\_2135 pos\_862 pos\_39 pos\_1982 pos\_752 pos\_766 pos\_2062 pos\_730 pos\_1044 pos\_4 pos\_1129 pos\_1844 pos\_671 pos\_57 pos\_220 pos\_36 pos\_2105 pos\_1442 pos\_754 |
| Digestive system | 04979 Cholesterol metabolism | 25 | pos\_10 pos\_1267 pos\_913 pos\_52 pos\_871 pos\_3 pos\_2730 pos\_879 pos\_1048 pos\_2854 pos\_2135 pos\_862 pos\_39 pos\_1982 pos\_752 pos\_766 pos\_2062 pos\_730 pos\_1044 pos\_4 pos\_1129 pos\_671 pos\_36 pos\_2105 pos\_1442 |
| Drug resistance: antineoplastic | 01521 EGFR tyrosine kinase inhibitor resistance | 1 | pos\_1442 |
| Drug resistance: antineoplastic | 01522 Endocrine resistance | 4 | pos\_1442 pos\_1165 pos\_1169 pos\_671 |
| Drug resistance: antineoplastic | 01523 Antifolate resistance | 2 | pos\_1442 pos\_671 |
| Drug resistance: antineoplastic | 01524 Platinum drug resistance | 1 | pos\_1442 |
| Endocrine and metabolic disease | 04931 Insulin resistance | 2 | pos\_1442 pos\_2759 |
| Endocrine and metabolic disease | 04933 AGE-RAGE signaling pathway in diabetic complications | 2 | pos\_1442 pos\_2759 |
| Endocrine and metabolic disease | 04934 Cushing syndrome | 2 | pos\_1442 pos\_2759 |
| Endocrine system | 03320 PPAR signaling pathway | 9 | pos\_1148 pos\_1442 pos\_5 pos\_1982 pos\_1025 pos\_9 pos\_879 pos\_614 pos\_1714 |
| Endocrine system | 04614 Renin-angiotensin system | 15 | pos\_545 pos\_1148 pos\_1835 pos\_1442 pos\_5 pos\_2072 pos\_1982 pos\_730 pos\_1165 pos\_1025 pos\_9 pos\_1169 pos\_879 pos\_614 pos\_1714 |
| Endocrine system | 04910 Insulin signaling pathway | 2 | pos\_1442 pos\_879 |
| Endocrine system | 04911 Insulin secretion | 2 | pos\_1442 pos\_879 |
| Endocrine system | 04912 GnRH signaling pathway | 11 | pos\_1148 pos\_1442 pos\_5 pos\_1982 pos\_1165 pos\_1025 pos\_9 pos\_1169 pos\_879 pos\_614 pos\_1714 |
| Endocrine system | 04913 Ovarian steroidogenesis | 13 | pos\_1148 pos\_1442 pos\_5 pos\_2072 pos\_1982 pos\_730 pos\_1165 pos\_1025 pos\_9 pos\_1169 pos\_879 pos\_614 pos\_1714 |
| Endocrine system | 04914 Progesterone-mediated oocyte maturation | 13 | pos\_1148 pos\_1442 pos\_5 pos\_2072 pos\_1982 pos\_730 pos\_1165 pos\_1025 pos\_9 pos\_1169 pos\_879 pos\_614 pos\_1714 |
| Endocrine system | 04915 Estrogen signaling pathway | 13 | pos\_1148 pos\_1442 pos\_5 pos\_2072 pos\_1982 pos\_730 pos\_1165 pos\_1025 pos\_9 pos\_1169 pos\_879 pos\_614 pos\_1714 |
| Endocrine system | 04916 Melanogenesis | 15 | pos\_545 pos\_1148 pos\_1835 pos\_1442 pos\_5 pos\_2072 pos\_1982 pos\_730 pos\_1165 pos\_1025 pos\_9 pos\_1169 pos\_879 pos\_614 pos\_1714 |
| Endocrine system | 04917 Prolactin signaling pathway | 13 | pos\_1148 pos\_1442 pos\_5 pos\_2072 pos\_1982 pos\_730 pos\_1165 pos\_1025 pos\_9 pos\_1169 pos\_879 pos\_614 pos\_1714 |
| Endocrine system | 04918 Thyroid hormone synthesis | 14 | pos\_545 pos\_1148 pos\_1442 pos\_5 pos\_2072 pos\_1982 pos\_730 pos\_1165 pos\_1025 pos\_9 pos\_1169 pos\_879 pos\_614 pos\_1714 |
| Endocrine system | 04919 Thyroid hormone signaling pathway | 14 | pos\_545 pos\_1148 pos\_1442 pos\_5 pos\_2072 pos\_1982 pos\_730 pos\_1165 pos\_1025 pos\_9 pos\_1169 pos\_879 pos\_614 pos\_1714 |
| Endocrine system | 04920 Adipocytokine signaling pathway | 9 | pos\_1148 pos\_1442 pos\_5 pos\_1982 pos\_1025 pos\_9 pos\_879 pos\_614 pos\_1714 |
| Endocrine system | 04921 Oxytocin signaling pathway | 13 | pos\_1148 pos\_1442 pos\_5 pos\_2072 pos\_1982 pos\_730 pos\_1165 pos\_1025 pos\_9 pos\_1169 pos\_879 pos\_614 pos\_1714 |
| Endocrine system | 04922 Glucagon signaling pathway | 4 | pos\_1442 pos\_5 pos\_879 pos\_614 |
| Endocrine system | 04923 Regulation of lipolysis in adipocytes | 8 | pos\_1148 pos\_1442 pos\_5 pos\_1982 pos\_1025 pos\_879 pos\_614 pos\_1714 |
| Endocrine system | 04924 Renin secretion | 15 | pos\_545 pos\_1148 pos\_1835 pos\_1442 pos\_5 pos\_2072 pos\_1982 pos\_730 pos\_1165 pos\_1025 pos\_9 pos\_1169 pos\_879 pos\_614 pos\_1714 |
| Endocrine system | 04925 Aldosterone synthesis and secretion | 16 | pos\_545 pos\_1148 pos\_1835 pos\_1442 pos\_5 pos\_2072 pos\_1982 pos\_730 pos\_1165 pos\_2601 pos\_1025 pos\_9 pos\_1169 pos\_879 pos\_614 pos\_1714 |
| Endocrine system | 04926 Relaxin signaling pathway | 13 | pos\_1148 pos\_1442 pos\_5 pos\_2072 pos\_1982 pos\_730 pos\_1165 pos\_1025 pos\_9 pos\_1169 pos\_879 pos\_614 pos\_1714 |
| Endocrine system | 04927 Cortisol synthesis and secretion | 16 | pos\_545 pos\_1148 pos\_1835 pos\_1442 pos\_5 pos\_2072 pos\_1982 pos\_730 pos\_1165 pos\_2601 pos\_1025 pos\_9 pos\_1169 pos\_879 pos\_614 pos\_1714 |
| Endocrine system | 04928 Parathyroid hormone synthesis, secretion and action | 15 | pos\_545 pos\_1148 pos\_1835 pos\_1442 pos\_5 pos\_2072 pos\_1982 pos\_730 pos\_1165 pos\_1025 pos\_9 pos\_1169 pos\_879 pos\_614 pos\_1714 |
| Endocrine system | 04929 GnRH secretion | 11 | pos\_1148 pos\_1442 pos\_5 pos\_1982 pos\_1165 pos\_1025 pos\_9 pos\_1169 pos\_879 pos\_614 pos\_1714 |
| Endocrine system | 04935 Growth hormone synthesis, secretion and action | 13 | pos\_1148 pos\_1442 pos\_5 pos\_2072 pos\_1982 pos\_730 pos\_1165 pos\_1025 pos\_9 pos\_1169 pos\_879 pos\_614 pos\_1714 |
| Energy metabolism | 00190 Oxidative phosphorylation | 3 | pos\_2601 pos\_2802 pos\_614 |
| Energy metabolism | 00195 Photosynthesis | 3 | pos\_2601 pos\_2802 pos\_614 |
| Energy metabolism | 00196 Photosynthesis - antenna proteins | 3 | pos\_2601 pos\_2802 pos\_614 |
| Energy metabolism | 00680 Methane metabolism | 3 | pos\_2601 pos\_2802 pos\_614 |
| Energy metabolism | 00710 Carbon fixation in photosynthetic organisms | 3 | pos\_2601 pos\_2802 pos\_614 |
| Energy metabolism | 00720 Carbon fixation pathways in prokaryotes | 3 | pos\_2601 pos\_2802 pos\_614 |
| Energy metabolism | 00910 Nitrogen metabolism | 3 | pos\_2601 pos\_2802 pos\_614 |
| Energy metabolism | 00920 Sulfur metabolism | 4 | pos\_2601 pos\_2802 pos\_614 pos\_2612 |
| Environmental adaptation | 04626 Plant-pathogen interaction | 4 | pos\_1442 pos\_1165 pos\_2601 pos\_884 |
| Environmental adaptation | 04711 Circadian rhythm - fly | 1 | pos\_1442 |
| Environmental adaptation | 04712 Circadian rhythm - plant | 1 | pos\_1442 |
| Environmental adaptation | 04713 Circadian entrainment | 1 | pos\_1442 |
| Environmental adaptation | 04714 Thermogenesis | 4 | pos\_1442 pos\_1165 pos\_2601 pos\_884 |
| Excretory system | 04961 Endocrine and other factor-regulated calcium reabsorption | 2 | pos\_1442 pos\_1165 |
| Excretory system | 04964 Proximal tubule bicarbonate reclamation | 2 | pos\_1442 pos\_1165 |
| Excretory system | 04966 Collecting duct acid secretion | 2 | pos\_1442 pos\_1165 |
| Global and overview maps | 01100 Metabolic pathways | 159 | pos\_735 pos\_2328 pos\_2601 pos\_3 pos\_759 pos\_663 pos\_1899 pos\_2 pos\_486 pos\_862 pos\_2805 pos\_1982 pos\_1746 pos\_2493 pos\_2853 pos\_709 pos\_1044 pos\_671 pos\_1018 pos\_1674 pos\_564 pos\_650 pos\_1740 pos\_577 pos\_36 pos\_1717 pos\_1007 pos\_1607 pos\_639 pos\_463 pos\_2279 pos\_13 pos\_1165 pos\_212 pos\_1148 pos\_809 pos\_1267 pos\_913 pos\_1241 pos\_856 pos\_1675 pos\_1050 pos\_2730 pos\_2768 pos\_388 pos\_2802 pos\_1783 pos\_1714 pos\_1048 pos\_2466 pos\_12 pos\_16 pos\_252 pos\_1817 pos\_975 pos\_2731 pos\_1806 pos\_2413 pos\_2844 pos\_766 pos\_1904 pos\_1370 pos\_474 pos\_1282 pos\_1709 pos\_1025 pos\_1129 pos\_9 pos\_1810 pos\_1 pos\_152 pos\_57 pos\_2166 pos\_784 pos\_2649 pos\_529 pos\_34 pos\_2218 pos\_1569 pos\_2612 pos\_813 pos\_2792 pos\_19 pos\_1986 pos\_52 pos\_1169 pos\_65 pos\_606 pos\_362 pos\_805 pos\_545 pos\_723 pos\_1195 pos\_2687 pos\_1110 pos\_39 pos\_1833 pos\_1264 pos\_331 pos\_126 pos\_2048 pos\_478 pos\_607 pos\_2274 pos\_1835 pos\_15 pos\_1430 pos\_4 pos\_2843 pos\_1860 pos\_556 pos\_220 pos\_1022 pos\_1517 pos\_869 pos\_754 pos\_1275 pos\_1955 pos\_911 pos\_2572 pos\_1752 pos\_2736 pos\_5 pos\_2409 pos\_53 pos\_2717 pos\_919 pos\_14 pos\_2785 pos\_75 pos\_2541 pos\_752 pos\_695 pos\_597 pos\_492 pos\_683 pos\_614 pos\_1898 pos\_835 pos\_1027 pos\_2054 pos\_1181 pos\_2734 pos\_431 pos\_457 pos\_1844 pos\_92 pos\_1745 pos\_2406 pos\_980 pos\_2105 pos\_2599 pos\_2850 pos\_1980 pos\_662 pos\_61 pos\_267 pos\_2727 pos\_112 |
| Global and overview maps | 01110 Biosynthesis of secondary metabolites | 159 | pos\_735 pos\_2328 pos\_2601 pos\_3 pos\_759 pos\_663 pos\_1899 pos\_2 pos\_486 pos\_862 pos\_2805 pos\_1982 pos\_1746 pos\_2493 pos\_2853 pos\_709 pos\_1044 pos\_671 pos\_1018 pos\_1674 pos\_564 pos\_650 pos\_1740 pos\_577 pos\_36 pos\_1717 pos\_1007 pos\_1607 pos\_639 pos\_463 pos\_2279 pos\_13 pos\_1165 pos\_212 pos\_1148 pos\_809 pos\_1267 pos\_913 pos\_1241 pos\_856 pos\_1675 pos\_1050 pos\_2730 pos\_2768 pos\_388 pos\_2802 pos\_1783 pos\_1714 pos\_1048 pos\_2466 pos\_12 pos\_16 pos\_252 pos\_1817 pos\_975 pos\_2731 pos\_1806 pos\_2413 pos\_2844 pos\_766 pos\_1904 pos\_1370 pos\_474 pos\_1282 pos\_1709 pos\_1025 pos\_1129 pos\_9 pos\_1810 pos\_1 pos\_152 pos\_57 pos\_2166 pos\_784 pos\_2649 pos\_529 pos\_34 pos\_2218 pos\_1569 pos\_2612 pos\_813 pos\_2792 pos\_19 pos\_1986 pos\_52 pos\_1169 pos\_65 pos\_606 pos\_362 pos\_805 pos\_545 pos\_723 pos\_1195 pos\_2687 pos\_1110 pos\_39 pos\_1833 pos\_1264 pos\_331 pos\_126 pos\_2048 pos\_478 pos\_607 pos\_2274 pos\_1835 pos\_15 pos\_1430 pos\_4 pos\_2843 pos\_1860 pos\_556 pos\_220 pos\_1022 pos\_1517 pos\_869 pos\_754 pos\_1275 pos\_1955 pos\_911 pos\_2572 pos\_1752 pos\_2736 pos\_5 pos\_2409 pos\_53 pos\_2717 pos\_919 pos\_14 pos\_2785 pos\_75 pos\_2541 pos\_752 pos\_695 pos\_597 pos\_492 pos\_683 pos\_614 pos\_1898 pos\_835 pos\_1027 pos\_2054 pos\_1181 pos\_2734 pos\_431 pos\_457 pos\_1844 pos\_92 pos\_1745 pos\_2406 pos\_980 pos\_2105 pos\_2599 pos\_2850 pos\_1980 pos\_662 pos\_61 pos\_267 pos\_2727 pos\_112 |
| Global and overview maps | 01120 Microbial metabolism in diverse environments | 159 | pos\_735 pos\_2328 pos\_2601 pos\_3 pos\_759 pos\_663 pos\_1899 pos\_2 pos\_486 pos\_862 pos\_2805 pos\_1982 pos\_1746 pos\_2493 pos\_2853 pos\_709 pos\_1044 pos\_671 pos\_1018 pos\_1674 pos\_564 pos\_650 pos\_1740 pos\_577 pos\_36 pos\_1717 pos\_1007 pos\_1607 pos\_639 pos\_463 pos\_2279 pos\_13 pos\_1165 pos\_212 pos\_1148 pos\_809 pos\_1267 pos\_913 pos\_1241 pos\_856 pos\_1675 pos\_1050 pos\_2730 pos\_2768 pos\_388 pos\_2802 pos\_1783 pos\_1714 pos\_1048 pos\_2466 pos\_12 pos\_16 pos\_252 pos\_1817 pos\_975 pos\_2731 pos\_1806 pos\_2413 pos\_2844 pos\_766 pos\_1904 pos\_1370 pos\_474 pos\_1282 pos\_1709 pos\_1025 pos\_1129 pos\_9 pos\_1810 pos\_1 pos\_152 pos\_57 pos\_2166 pos\_784 pos\_2649 pos\_529 pos\_34 pos\_2218 pos\_1569 pos\_2612 pos\_813 pos\_2792 pos\_19 pos\_1986 pos\_52 pos\_1169 pos\_65 pos\_606 pos\_362 pos\_805 pos\_545 pos\_723 pos\_1195 pos\_2687 pos\_1110 pos\_39 pos\_1833 pos\_1264 pos\_331 pos\_126 pos\_2048 pos\_478 pos\_607 pos\_2274 pos\_1835 pos\_15 pos\_1430 pos\_4 pos\_2843 pos\_1860 pos\_556 pos\_220 pos\_1022 pos\_1517 pos\_869 pos\_754 pos\_1275 pos\_1955 pos\_911 pos\_2572 pos\_1752 pos\_2736 pos\_5 pos\_2409 pos\_53 pos\_2717 pos\_919 pos\_14 pos\_2785 pos\_75 pos\_2541 pos\_752 pos\_695 pos\_597 pos\_492 pos\_683 pos\_614 pos\_1898 pos\_835 pos\_1027 pos\_2054 pos\_1181 pos\_2734 pos\_431 pos\_457 pos\_1844 pos\_92 pos\_1745 pos\_2406 pos\_980 pos\_2105 pos\_2599 pos\_2850 pos\_1980 pos\_662 pos\_61 pos\_267 pos\_2727 pos\_112 |
| Global and overview maps | 01200 Carbon metabolism | 159 | pos\_735 pos\_2328 pos\_2601 pos\_3 pos\_759 pos\_663 pos\_1899 pos\_2 pos\_486 pos\_862 pos\_2805 pos\_1982 pos\_1746 pos\_2493 pos\_2853 pos\_709 pos\_1044 pos\_671 pos\_1018 pos\_1674 pos\_564 pos\_650 pos\_1740 pos\_577 pos\_36 pos\_1717 pos\_1007 pos\_1607 pos\_639 pos\_463 pos\_2279 pos\_13 pos\_1165 pos\_212 pos\_1148 pos\_809 pos\_1267 pos\_913 pos\_1241 pos\_856 pos\_1675 pos\_1050 pos\_2730 pos\_2768 pos\_388 pos\_2802 pos\_1783 pos\_1714 pos\_1048 pos\_2466 pos\_12 pos\_16 pos\_252 pos\_1817 pos\_975 pos\_2731 pos\_1806 pos\_2413 pos\_2844 pos\_766 pos\_1904 pos\_1370 pos\_474 pos\_1282 pos\_1709 pos\_1025 pos\_1129 pos\_9 pos\_1810 pos\_1 pos\_152 pos\_57 pos\_2166 pos\_784 pos\_2649 pos\_529 pos\_34 pos\_2218 pos\_1569 pos\_2612 pos\_813 pos\_2792 pos\_19 pos\_1986 pos\_52 pos\_1169 pos\_65 pos\_606 pos\_362 pos\_805 pos\_545 pos\_723 pos\_1195 pos\_2687 pos\_1110 pos\_39 pos\_1833 pos\_1264 pos\_331 pos\_126 pos\_2048 pos\_478 pos\_607 pos\_2274 pos\_1835 pos\_15 pos\_1430 pos\_4 pos\_2843 pos\_1860 pos\_556 pos\_220 pos\_1022 pos\_1517 pos\_869 pos\_754 pos\_1275 pos\_1955 pos\_911 pos\_2572 pos\_1752 pos\_2736 pos\_5 pos\_2409 pos\_53 pos\_2717 pos\_919 pos\_14 pos\_2785 pos\_75 pos\_2541 pos\_752 pos\_695 pos\_597 pos\_492 pos\_683 pos\_614 pos\_1898 pos\_835 pos\_1027 pos\_2054 pos\_1181 pos\_2734 pos\_431 pos\_457 pos\_1844 pos\_92 pos\_1745 pos\_2406 pos\_980 pos\_2105 pos\_2599 pos\_2850 pos\_1980 pos\_662 pos\_61 pos\_267 pos\_2727 pos\_112 |
| Global and overview maps | 01210 2-Oxocarboxylic acid metabolism | 161 | pos\_735 pos\_2328 pos\_2601 pos\_3 pos\_759 pos\_663 pos\_1899 pos\_2 pos\_486 pos\_862 pos\_2433 pos\_2805 pos\_1982 pos\_1746 pos\_2493 pos\_2853 pos\_709 pos\_1044 pos\_671 pos\_1018 pos\_1674 pos\_564 pos\_650 pos\_1740 pos\_577 pos\_36 pos\_1717 pos\_1007 pos\_1607 pos\_639 pos\_463 pos\_2279 pos\_13 pos\_1165 pos\_212 pos\_1148 pos\_809 pos\_1267 pos\_913 pos\_1241 pos\_856 pos\_1675 pos\_1050 pos\_2730 pos\_2768 pos\_388 pos\_2802 pos\_1783 pos\_1714 pos\_1048 pos\_2466 pos\_12 pos\_16 pos\_252 pos\_1817 pos\_975 pos\_2731 pos\_1806 pos\_2413 pos\_2844 pos\_766 pos\_1904 pos\_1370 pos\_474 pos\_1282 pos\_1709 pos\_1025 pos\_1129 pos\_9 pos\_1810 pos\_1 pos\_152 pos\_57 pos\_2166 pos\_784 pos\_2649 pos\_529 pos\_34 pos\_2218 pos\_1569 pos\_2612 pos\_813 pos\_2792 pos\_19 pos\_1986 pos\_52 pos\_1169 pos\_65 pos\_606 pos\_362 pos\_805 pos\_545 pos\_723 pos\_1195 pos\_2687 pos\_1110 pos\_39 pos\_1833 pos\_1264 pos\_331 pos\_126 pos\_2048 pos\_478 pos\_607 pos\_2274 pos\_1835 pos\_15 pos\_1430 pos\_4 pos\_2843 pos\_1860 pos\_556 pos\_220 pos\_1022 pos\_1517 pos\_869 pos\_754 pos\_1275 pos\_1955 pos\_911 pos\_2572 pos\_1752 pos\_2736 pos\_5 pos\_2409 pos\_53 pos\_2717 pos\_919 pos\_14 pos\_2785 pos\_75 pos\_2541 pos\_752 pos\_695 pos\_597 pos\_492 pos\_683 pos\_614 pos\_1898 pos\_835 pos\_1027 pos\_2054 pos\_1181 pos\_2734 pos\_431 pos\_457 pos\_1315 pos\_1844 pos\_92 pos\_1745 pos\_2406 pos\_980 pos\_2105 pos\_2599 pos\_2850 pos\_1980 pos\_662 pos\_61 pos\_267 pos\_2727 pos\_112 |
| Global and overview maps | 01212 Fatty acid metabolism | 162 | pos\_735 pos\_2328 pos\_2601 pos\_3 pos\_759 pos\_663 pos\_1899 pos\_2 pos\_486 pos\_862 pos\_2433 pos\_2805 pos\_1982 pos\_1746 pos\_2493 pos\_2853 pos\_2744 pos\_709 pos\_1044 pos\_671 pos\_1018 pos\_1674 pos\_564 pos\_650 pos\_1740 pos\_577 pos\_36 pos\_1717 pos\_1007 pos\_1607 pos\_639 pos\_463 pos\_2279 pos\_13 pos\_1165 pos\_212 pos\_1148 pos\_809 pos\_1267 pos\_913 pos\_1241 pos\_856 pos\_1675 pos\_1050 pos\_2730 pos\_2768 pos\_388 pos\_2802 pos\_1783 pos\_1714 pos\_1048 pos\_2466 pos\_12 pos\_16 pos\_252 pos\_1817 pos\_975 pos\_2731 pos\_1806 pos\_2413 pos\_2844 pos\_766 pos\_1904 pos\_1370 pos\_474 pos\_1282 pos\_1709 pos\_1025 pos\_1129 pos\_9 pos\_1810 pos\_1 pos\_152 pos\_57 pos\_2166 pos\_784 pos\_2649 pos\_529 pos\_34 pos\_2218 pos\_1569 pos\_2612 pos\_813 pos\_2792 pos\_19 pos\_1986 pos\_52 pos\_1169 pos\_65 pos\_606 pos\_362 pos\_805 pos\_545 pos\_723 pos\_1195 pos\_2687 pos\_1110 pos\_39 pos\_1833 pos\_1264 pos\_331 pos\_126 pos\_2048 pos\_478 pos\_607 pos\_2274 pos\_1835 pos\_15 pos\_1430 pos\_4 pos\_2843 pos\_1860 pos\_556 pos\_220 pos\_1022 pos\_1517 pos\_869 pos\_754 pos\_1275 pos\_1955 pos\_911 pos\_2572 pos\_1752 pos\_2736 pos\_5 pos\_2409 pos\_53 pos\_2717 pos\_919 pos\_14 pos\_2785 pos\_75 pos\_2541 pos\_752 pos\_695 pos\_597 pos\_492 pos\_683 pos\_614 pos\_1898 pos\_835 pos\_1027 pos\_2054 pos\_1181 pos\_2734 pos\_431 pos\_457 pos\_1315 pos\_1844 pos\_92 pos\_1745 pos\_2406 pos\_980 pos\_2105 pos\_2599 pos\_2850 pos\_1980 pos\_662 pos\_61 pos\_267 pos\_2727 pos\_112 |
| Global and overview maps | 01220 Degradation of aromatic compounds | 162 | pos\_735 pos\_2328 pos\_2601 pos\_3 pos\_759 pos\_663 pos\_1899 pos\_2 pos\_486 pos\_862 pos\_2433 pos\_2805 pos\_1982 pos\_1746 pos\_2493 pos\_2853 pos\_2744 pos\_709 pos\_1044 pos\_671 pos\_1018 pos\_1674 pos\_564 pos\_650 pos\_1740 pos\_577 pos\_36 pos\_1717 pos\_1007 pos\_1607 pos\_639 pos\_463 pos\_2279 pos\_13 pos\_1165 pos\_212 pos\_1148 pos\_809 pos\_1267 pos\_913 pos\_1241 pos\_856 pos\_1675 pos\_1050 pos\_2730 pos\_2768 pos\_388 pos\_2802 pos\_1783 pos\_1714 pos\_1048 pos\_2466 pos\_12 pos\_16 pos\_252 pos\_1817 pos\_975 pos\_2731 pos\_1806 pos\_2413 pos\_2844 pos\_766 pos\_1904 pos\_1370 pos\_474 pos\_1282 pos\_1709 pos\_1025 pos\_1129 pos\_9 pos\_1810 pos\_1 pos\_152 pos\_57 pos\_2166 pos\_784 pos\_2649 pos\_529 pos\_34 pos\_2218 pos\_1569 pos\_2612 pos\_813 pos\_2792 pos\_19 pos\_1986 pos\_52 pos\_1169 pos\_65 pos\_606 pos\_362 pos\_805 pos\_545 pos\_723 pos\_1195 pos\_2687 pos\_1110 pos\_39 pos\_1833 pos\_1264 pos\_331 pos\_126 pos\_2048 pos\_478 pos\_607 pos\_2274 pos\_1835 pos\_15 pos\_1430 pos\_4 pos\_2843 pos\_1860 pos\_556 pos\_220 pos\_1022 pos\_1517 pos\_869 pos\_754 pos\_1275 pos\_1955 pos\_911 pos\_2572 pos\_1752 pos\_2736 pos\_5 pos\_2409 pos\_53 pos\_2717 pos\_919 pos\_14 pos\_2785 pos\_75 pos\_2541 pos\_752 pos\_695 pos\_597 pos\_492 pos\_683 pos\_614 pos\_1898 pos\_835 pos\_1027 pos\_2054 pos\_1181 pos\_2734 pos\_431 pos\_457 pos\_1315 pos\_1844 pos\_92 pos\_1745 pos\_2406 pos\_980 pos\_2105 pos\_2599 pos\_2850 pos\_1980 pos\_662 pos\_61 pos\_267 pos\_2727 pos\_112 |
| Global and overview maps | 01230 Biosynthesis of amino acids | 162 | pos\_735 pos\_2328 pos\_2601 pos\_3 pos\_759 pos\_663 pos\_1899 pos\_2 pos\_486 pos\_862 pos\_2433 pos\_2805 pos\_1982 pos\_1746 pos\_2493 pos\_2853 pos\_2744 pos\_709 pos\_1044 pos\_671 pos\_1018 pos\_1674 pos\_564 pos\_650 pos\_1740 pos\_577 pos\_36 pos\_1717 pos\_1007 pos\_1607 pos\_639 pos\_463 pos\_2279 pos\_13 pos\_1165 pos\_212 pos\_1148 pos\_809 pos\_1267 pos\_913 pos\_1241 pos\_856 pos\_1675 pos\_1050 pos\_2730 pos\_2768 pos\_388 pos\_2802 pos\_1783 pos\_1714 pos\_1048 pos\_2466 pos\_12 pos\_16 pos\_252 pos\_1817 pos\_975 pos\_2731 pos\_1806 pos\_2413 pos\_2844 pos\_766 pos\_1904 pos\_1370 pos\_474 pos\_1282 pos\_1709 pos\_1025 pos\_1129 pos\_9 pos\_1810 pos\_1 pos\_152 pos\_57 pos\_2166 pos\_784 pos\_2649 pos\_529 pos\_34 pos\_2218 pos\_1569 pos\_2612 pos\_813 pos\_2792 pos\_19 pos\_1986 pos\_52 pos\_1169 pos\_65 pos\_606 pos\_362 pos\_805 pos\_545 pos\_723 pos\_1195 pos\_2687 pos\_1110 pos\_39 pos\_1833 pos\_1264 pos\_331 pos\_126 pos\_2048 pos\_478 pos\_607 pos\_2274 pos\_1835 pos\_15 pos\_1430 pos\_4 pos\_2843 pos\_1860 pos\_556 pos\_220 pos\_1022 pos\_1517 pos\_869 pos\_754 pos\_1275 pos\_1955 pos\_911 pos\_2572 pos\_1752 pos\_2736 pos\_5 pos\_2409 pos\_53 pos\_2717 pos\_919 pos\_14 pos\_2785 pos\_75 pos\_2541 pos\_752 pos\_695 pos\_597 pos\_492 pos\_683 pos\_614 pos\_1898 pos\_835 pos\_1027 pos\_2054 pos\_1181 pos\_2734 pos\_431 pos\_457 pos\_1315 pos\_1844 pos\_92 pos\_1745 pos\_2406 pos\_980 pos\_2105 pos\_2599 pos\_2850 pos\_1980 pos\_662 pos\_61 pos\_267 pos\_2727 pos\_112 |
| Global and overview maps | 01240 Biosynthesis of cofactors | 162 | pos\_735 pos\_2328 pos\_2601 pos\_3 pos\_759 pos\_663 pos\_1899 pos\_2 pos\_486 pos\_862 pos\_2433 pos\_2805 pos\_1982 pos\_1746 pos\_2493 pos\_2853 pos\_2744 pos\_709 pos\_1044 pos\_671 pos\_1018 pos\_1674 pos\_564 pos\_650 pos\_1740 pos\_577 pos\_36 pos\_1717 pos\_1007 pos\_1607 pos\_639 pos\_463 pos\_2279 pos\_13 pos\_1165 pos\_212 pos\_1148 pos\_809 pos\_1267 pos\_913 pos\_1241 pos\_856 pos\_1675 pos\_1050 pos\_2730 pos\_2768 pos\_388 pos\_2802 pos\_1783 pos\_1714 pos\_1048 pos\_2466 pos\_12 pos\_16 pos\_252 pos\_1817 pos\_975 pos\_2731 pos\_1806 pos\_2413 pos\_2844 pos\_766 pos\_1904 pos\_1370 pos\_474 pos\_1282 pos\_1709 pos\_1025 pos\_1129 pos\_9 pos\_1810 pos\_1 pos\_152 pos\_57 pos\_2166 pos\_784 pos\_2649 pos\_529 pos\_34 pos\_2218 pos\_1569 pos\_2612 pos\_813 pos\_2792 pos\_19 pos\_1986 pos\_52 pos\_1169 pos\_65 pos\_606 pos\_362 pos\_805 pos\_545 pos\_723 pos\_1195 pos\_2687 pos\_1110 pos\_39 pos\_1833 pos\_1264 pos\_331 pos\_126 pos\_2048 pos\_478 pos\_607 pos\_2274 pos\_1835 pos\_15 pos\_1430 pos\_4 pos\_2843 pos\_1860 pos\_556 pos\_220 pos\_1022 pos\_1517 pos\_869 pos\_754 pos\_1275 pos\_1955 pos\_911 pos\_2572 pos\_1752 pos\_2736 pos\_5 pos\_2409 pos\_53 pos\_2717 pos\_919 pos\_14 pos\_2785 pos\_75 pos\_2541 pos\_752 pos\_695 pos\_597 pos\_492 pos\_683 pos\_614 pos\_1898 pos\_835 pos\_1027 pos\_2054 pos\_1181 pos\_2734 pos\_431 pos\_457 pos\_1315 pos\_1844 pos\_92 pos\_1745 pos\_2406 pos\_980 pos\_2105 pos\_2599 pos\_2850 pos\_1980 pos\_662 pos\_61 pos\_267 pos\_2727 pos\_112 |
| Glycan biosynthesis and metabolism | 00510 N-Glycan biosynthesis | 1 | pos\_1282 |
| Glycan biosynthesis and metabolism | 00511 Other glycan degradation | 4 | pos\_1282 pos\_1007 pos\_331 pos\_2649 |
| Glycan biosynthesis and metabolism | 00512 Mucin type O-glycan biosynthesis | 1 | pos\_1282 |
| Glycan biosynthesis and metabolism | 00513 Various types of N-glycan biosynthesis | 1 | pos\_1282 |
| Glycan biosynthesis and metabolism | 00514 Other types of O-glycan biosynthesis | 1 | pos\_1282 |
| Glycan biosynthesis and metabolism | 00515 Mannose type O-glycan biosynthesis | 1 | pos\_1282 |
| Glycan biosynthesis and metabolism | 00531 Glycosaminoglycan degradation | 2 | pos\_1282 pos\_2649 |
| Glycan biosynthesis and metabolism | 00532 Glycosaminoglycan biosynthesis - chondroitin sulfate / dermatan sulfate | 1 | pos\_1282 |
| Glycan biosynthesis and metabolism | 00533 Glycosaminoglycan biosynthesis - keratan sulfate | 1 | pos\_1282 |
| Glycan biosynthesis and metabolism | 00534 Glycosaminoglycan biosynthesis - heparan sulfate / heparin | 1 | pos\_1282 |
| Glycan biosynthesis and metabolism | 00540 Lipopolysaccharide biosynthesis | 4 | pos\_1282 pos\_1007 pos\_331 pos\_2649 |
| Glycan biosynthesis and metabolism | 00541 O-Antigen nucleotide sugar biosynthesis | 4 | pos\_1282 pos\_1007 pos\_331 pos\_2649 |
| Glycan biosynthesis and metabolism | 00542 O-Antigen repeat unit biosynthesis | 4 | pos\_1282 pos\_1007 pos\_331 pos\_2649 |
| Glycan biosynthesis and metabolism | 00550 Peptidoglycan biosynthesis | 4 | pos\_1282 pos\_1007 pos\_331 pos\_2649 |
| Glycan biosynthesis and metabolism | 00563 Glycosylphosphatidylinositol (GPI)-anchor biosynthesis | 4 | pos\_1282 pos\_1007 pos\_331 pos\_2649 |
| Glycan biosynthesis and metabolism | 00571 Lipoarabinomannan (LAM) biosynthesis | 4 | pos\_1282 pos\_1007 pos\_331 pos\_2649 |
| Glycan biosynthesis and metabolism | 00572 Arabinogalactan biosynthesis - Mycobacterium | 4 | pos\_1282 pos\_1007 pos\_331 pos\_2649 |
| Glycan biosynthesis and metabolism | 00601 Glycosphingolipid biosynthesis - lacto and neolacto series | 4 | pos\_1282 pos\_1007 pos\_331 pos\_2649 |
| Glycan biosynthesis and metabolism | 00603 Glycosphingolipid biosynthesis - globo and isoglobo series | 4 | pos\_1282 pos\_1007 pos\_331 pos\_2649 |
| Glycan biosynthesis and metabolism | 00604 Glycosphingolipid biosynthesis - ganglio series | 4 | pos\_1282 pos\_1007 pos\_331 pos\_2649 |
| Immune disease | 05310 Asthma | 1 | pos\_2730 |
| Immune disease | 05320 Autoimmune thyroid disease | 4 | pos\_2730 pos\_57 pos\_1982 pos\_1898 |
| Immune disease | 05321 Inflammatory bowel disease | 4 | pos\_2730 pos\_57 pos\_1982 pos\_1898 |
| Immune disease | 05322 Systemic lupus erythematosus | 2 | pos\_2730 pos\_1898 |
| Immune disease | 05323 Rheumatoid arthritis | 4 | pos\_2730 pos\_57 pos\_1982 pos\_1898 |
| Immune disease | 05330 Allograft rejection | 4 | pos\_2730 pos\_57 pos\_1982 pos\_1898 |
| Immune disease | 05332 Graft-versus-host disease | 4 | pos\_2730 pos\_57 pos\_1982 pos\_1898 |
| Immune disease | 05340 Primary immunodeficiency | 4 | pos\_2730 pos\_57 pos\_1982 pos\_1898 |
| Immune system | 04062 Chemokine signaling pathway | 6 | pos\_1442 pos\_662 pos\_1982 pos\_9 pos\_2730 pos\_1714 |
| Immune system | 04611 Platelet activation | 2 | pos\_1442 pos\_1714 |
| Immune system | 04612 Antigen processing and presentation | 3 | pos\_1442 pos\_1982 pos\_1714 |
| Immune system | 04613 Neutrophil extracellular trap formation | 2 | pos\_1442 pos\_1714 |
| Immune system | 04620 Toll-like receptor signaling pathway | 2 | pos\_1442 pos\_1714 |
| Immune system | 04621 NOD-like receptor signaling pathway | 2 | pos\_1442 pos\_1714 |
| Immune system | 04622 RIG-I-like receptor signaling pathway | 2 | pos\_1442 pos\_1714 |
| Immune system | 04623 Cytosolic DNA-sensing pathway | 2 | pos\_1442 pos\_1714 |
| Immune system | 04624 Toll and Imd signaling pathway | 2 | pos\_1442 pos\_1714 |
| Immune system | 04625 C-type lectin receptor signaling pathway | 3 | pos\_1442 pos\_1982 pos\_1714 |
| Immune system | 04650 Natural killer cell mediated cytotoxicity | 3 | pos\_1442 pos\_1982 pos\_1714 |
| Immune system | 04657 IL-17 signaling pathway | 5 | pos\_1442 pos\_662 pos\_1982 pos\_9 pos\_1714 |
| Immune system | 04658 Th1 and Th2 cell differentiation | 3 | pos\_1442 pos\_1982 pos\_1714 |
| Immune system | 04659 Th17 cell differentiation | 5 | pos\_1442 pos\_662 pos\_1982 pos\_9 pos\_1714 |
| Immune system | 04660 T cell receptor signaling pathway | 3 | pos\_1442 pos\_1982 pos\_1714 |
| Immune system | 04662 B cell receptor signaling pathway | 5 | pos\_1442 pos\_662 pos\_1982 pos\_9 pos\_1714 |
| Immune system | 04664 Fc epsilon RI signaling pathway | 6 | pos\_1442 pos\_662 pos\_1982 pos\_9 pos\_2730 pos\_1714 |
| Immune system | 04666 Fc gamma R-mediated phagocytosis | 6 | pos\_1442 pos\_662 pos\_1982 pos\_9 pos\_2730 pos\_1714 |
| Immune system | 04670 Leukocyte transendothelial migration | 6 | pos\_1442 pos\_662 pos\_1982 pos\_9 pos\_2730 pos\_1714 |
| Immune system | 04672 Intestinal immune network for IgA production | 6 | pos\_1442 pos\_662 pos\_1982 pos\_9 pos\_2730 pos\_1714 |
| Infectious disease: bacterial | 05100 Bacterial invasion of epithelial cells | 5 | pos\_1835 pos\_1007 pos\_1442 pos\_1044 pos\_331 |
| Infectious disease: bacterial | 05110 Vibrio cholerae infection | 1 | pos\_1442 |
| Infectious disease: bacterial | 05120 Epithelial cell signaling in Helicobacter pylori infection | 1 | pos\_1442 |
| Infectious disease: bacterial | 05130 Pathogenic Escherichia coli infection | 3 | pos\_1442 pos\_1007 pos\_331 |
| Infectious disease: bacterial | 05131 Shigellosis | 4 | pos\_1442 pos\_1007 pos\_1044 pos\_331 |
| Infectious disease: bacterial | 05132 Salmonella infection | 3 | pos\_1442 pos\_1007 pos\_331 |
| Infectious disease: bacterial | 05133 Pertussis | 4 | pos\_1442 pos\_1007 pos\_1044 pos\_331 |
| Infectious disease: bacterial | 05134 Legionellosis | 4 | pos\_1442 pos\_1007 pos\_1044 pos\_331 |
| Infectious disease: bacterial | 05135 Yersinia infection | 4 | pos\_1442 pos\_1007 pos\_1044 pos\_331 |
| Infectious disease: bacterial | 05150 Staphylococcus aureus infection | 4 | pos\_1442 pos\_1007 pos\_1044 pos\_331 |
| Infectious disease: bacterial | 05152 Tuberculosis | 5 | pos\_1835 pos\_1007 pos\_1442 pos\_1044 pos\_331 |
| Infectious disease: parasitic | 05140 Leishmaniasis | 6 | pos\_766 pos\_2649 pos\_1442 pos\_1714 pos\_1982 pos\_1898 |
| Infectious disease: parasitic | 05142 Chagas disease | 6 | pos\_766 pos\_2649 pos\_1442 pos\_1714 pos\_1982 pos\_1898 |
| Infectious disease: parasitic | 05143 African trypanosomiasis | 6 | pos\_766 pos\_2649 pos\_1442 pos\_1714 pos\_1982 pos\_1898 |
| Infectious disease: parasitic | 05144 Malaria | 6 | pos\_766 pos\_2649 pos\_1442 pos\_1714 pos\_1982 pos\_1898 |
| Infectious disease: parasitic | 05145 Toxoplasmosis | 6 | pos\_766 pos\_2649 pos\_1442 pos\_1714 pos\_1982 pos\_1898 |
| Infectious disease: parasitic | 05146 Amoebiasis | 5 | pos\_766 pos\_1442 pos\_1714 pos\_1982 pos\_1898 |
| Infectious disease: viral | 05160 Hepatitis C | 1 | pos\_1442 |
| Infectious disease: viral | 05161 Hepatitis B | 1 | pos\_1442 |
| Infectious disease: viral | 05162 Measles | 1 | pos\_1442 |
| Infectious disease: viral | 05163 Human cytomegalovirus infection | 2 | pos\_1442 pos\_1982 |
| Infectious disease: viral | 05164 Influenza A | 1 | pos\_1442 |
| Infectious disease: viral | 05165 Human papillomavirus infection | 4 | pos\_1442 pos\_1982 pos\_1007 pos\_331 |
| Infectious disease: viral | 05167 Kaposi sarcoma-associated herpesvirus infection | 4 | pos\_1442 pos\_1982 pos\_1007 pos\_331 |
| Infectious disease: viral | 05168 Herpes simplex virus 1 infection | 1 | pos\_1442 |
| Infectious disease: viral | 05169 Epstein-Barr virus infection | 4 | pos\_1442 pos\_1982 pos\_1007 pos\_331 |
| Infectious disease: viral | 05170 Human immunodeficiency virus 1 infection | 1 | pos\_1442 |
| Infectious disease: viral | 05171 Coronavirus disease - COVID-19 | 1 | pos\_1442 |
| Lipid metabolism | 00061 Fatty acid biosynthesis | 5 | pos\_14 pos\_499 pos\_478 pos\_1355 pos\_663 |
| Lipid metabolism | 00062 Fatty acid elongation | 5 | pos\_14 pos\_499 pos\_478 pos\_1355 pos\_663 |
| Lipid metabolism | 00071 Fatty acid degradation | 6 | pos\_14 pos\_2744 pos\_499 pos\_478 pos\_1355 pos\_663 |
| Lipid metabolism | 00072 Synthesis and degradation of ketone bodies | 6 | pos\_14 pos\_2744 pos\_499 pos\_478 pos\_1355 pos\_663 |
| Lipid metabolism | 00073 Cutin, suberine and wax biosynthesis | 6 | pos\_14 pos\_2744 pos\_499 pos\_478 pos\_1355 pos\_663 |
| Lipid metabolism | 00100 Steroid biosynthesis | 12 | pos\_919 pos\_1835 pos\_14 pos\_1783 pos\_2744 pos\_2054 pos\_730 pos\_499 pos\_478 pos\_1355 pos\_663 pos\_57 |
| Lipid metabolism | 00120 Primary bile acid biosynthesis | 14 | pos\_919 pos\_1835 pos\_14 pos\_1783 pos\_2744 pos\_2105 pos\_2054 pos\_730 pos\_499 pos\_3 pos\_478 pos\_1355 pos\_663 pos\_57 |
| Lipid metabolism | 00121 Secondary bile acid biosynthesis | 14 | pos\_919 pos\_1835 pos\_14 pos\_1783 pos\_2744 pos\_2105 pos\_2054 pos\_730 pos\_499 pos\_3 pos\_478 pos\_1355 pos\_663 pos\_57 |
| Lipid metabolism | 00140 Steroid hormone biosynthesis | 21 | pos\_1148 pos\_3 pos\_1169 pos\_1407 pos\_1783 pos\_663 pos\_919 pos\_14 pos\_2072 pos\_1204 pos\_478 pos\_1835 pos\_2744 pos\_2054 pos\_730 pos\_499 pos\_507 pos\_1355 pos\_57 pos\_2105 pos\_1165 |
| Lipid metabolism | 00561 Glycerolipid metabolism | 22 | pos\_1148 pos\_3 pos\_1169 pos\_1407 pos\_1783 pos\_663 pos\_919 pos\_14 pos\_2072 pos\_1204 pos\_478 pos\_1835 pos\_2744 pos\_2054 pos\_730 pos\_499 pos\_1087 pos\_507 pos\_1355 pos\_57 pos\_2105 pos\_1165 |
| Lipid metabolism | 00564 Glycerophospholipid metabolism | 35 | pos\_1148 pos\_3 pos\_1169 pos\_879 pos\_1407 pos\_1783 pos\_663 pos\_2204 pos\_919 pos\_14 pos\_2072 pos\_1833 pos\_39 pos\_2769 pos\_331 pos\_1806 pos\_1204 pos\_478 pos\_1898 pos\_1835 pos\_2744 pos\_2054 pos\_730 pos\_499 pos\_1430 pos\_1018 pos\_1087 pos\_507 pos\_2263 pos\_1355 pos\_57 pos\_2105 pos\_1007 pos\_1165 pos\_1619 |
| Lipid metabolism | 00565 Ether lipid metabolism | 35 | pos\_1148 pos\_3 pos\_1169 pos\_879 pos\_1407 pos\_1783 pos\_663 pos\_2204 pos\_919 pos\_14 pos\_2072 pos\_1833 pos\_39 pos\_2769 pos\_331 pos\_1806 pos\_1204 pos\_478 pos\_1898 pos\_1835 pos\_2744 pos\_2054 pos\_730 pos\_499 pos\_1430 pos\_1018 pos\_1087 pos\_507 pos\_2263 pos\_1355 pos\_57 pos\_2105 pos\_1007 pos\_1165 pos\_1619 |
| Lipid metabolism | 00590 Arachidonic acid metabolism | 47 | pos\_1148 pos\_3 pos\_1169 pos\_404 pos\_879 pos\_1407 pos\_1783 pos\_663 pos\_2204 pos\_919 pos\_1714 pos\_723 pos\_14 pos\_1110 pos\_2072 pos\_1833 pos\_39 pos\_1982 pos\_2769 pos\_331 pos\_1806 pos\_1204 pos\_478 pos\_607 pos\_1898 pos\_1835 pos\_2744 pos\_2054 pos\_730 pos\_499 pos\_1430 pos\_1709 pos\_1018 pos\_1087 pos\_507 pos\_2263 pos\_1355 pos\_57 pos\_1 pos\_577 pos\_1019 pos\_2105 pos\_1007 pos\_2166 pos\_1165 pos\_1619 pos\_813 |
| Lipid metabolism | 00591 Linoleic acid metabolism | 51 | pos\_1148 pos\_3 pos\_1169 pos\_404 pos\_879 pos\_1407 pos\_1783 pos\_663 pos\_2204 pos\_919 pos\_1714 pos\_723 pos\_1005 pos\_14 pos\_1110 pos\_2072 pos\_1833 pos\_39 pos\_1982 pos\_2769 pos\_695 pos\_331 pos\_1806 pos\_1204 pos\_478 pos\_607 pos\_1898 pos\_1835 pos\_2744 pos\_1904 pos\_2054 pos\_730 pos\_499 pos\_1430 pos\_1709 pos\_1018 pos\_1087 pos\_507 pos\_2263 pos\_1355 pos\_57 pos\_1 pos\_577 pos\_1019 pos\_2105 pos\_1007 pos\_2166 pos\_1980 pos\_1165 pos\_1619 pos\_813 |
| Lipid metabolism | 00592 alpha-Linolenic acid metabolism | 59 | pos\_1148 pos\_659 pos\_161 pos\_3 pos\_1169 pos\_404 pos\_879 pos\_1407 pos\_1783 pos\_663 pos\_2204 pos\_919 pos\_1714 pos\_723 pos\_1005 pos\_14 pos\_1110 pos\_560 pos\_2072 pos\_1833 pos\_39 pos\_1982 pos\_2769 pos\_695 pos\_331 pos\_1806 pos\_2812 pos\_1204 pos\_478 pos\_607 pos\_1898 pos\_1835 pos\_2744 pos\_1904 pos\_2054 pos\_730 pos\_499 pos\_1430 pos\_1709 pos\_101 pos\_1018 pos\_1087 pos\_507 pos\_2263 pos\_1355 pos\_57 pos\_1 pos\_83 pos\_577 pos\_1019 pos\_2105 pos\_1007 pos\_2166 pos\_463 pos\_1980 pos\_1165 pos\_1619 pos\_813 pos\_2727 |
| Lipid metabolism | 00600 Sphingolipid metabolism | 38 | pos\_1148 pos\_3 pos\_1169 pos\_879 pos\_1407 pos\_1783 pos\_663 pos\_2204 pos\_919 pos\_14 pos\_2072 pos\_1833 pos\_39 pos\_2769 pos\_331 pos\_1806 pos\_1204 pos\_478 pos\_1898 pos\_1835 pos\_2744 pos\_2054 pos\_730 pos\_499 pos\_1430 pos\_1709 pos\_1018 pos\_1087 pos\_507 pos\_2263 pos\_1355 pos\_57 pos\_1 pos\_2105 pos\_1007 pos\_1165 pos\_1619 pos\_813 |
| Lipid metabolism | 01040 Biosynthesis of unsaturated fatty acids | 64 | pos\_1148 pos\_659 pos\_161 pos\_1252 pos\_3 pos\_1169 pos\_404 pos\_879 pos\_1407 pos\_1783 pos\_663 pos\_2204 pos\_919 pos\_1714 pos\_723 pos\_1005 pos\_522 pos\_14 pos\_1110 pos\_560 pos\_2072 pos\_1833 pos\_39 pos\_1982 pos\_2769 pos\_695 pos\_331 pos\_1806 pos\_2812 pos\_1204 pos\_478 pos\_607 pos\_1898 pos\_1835 pos\_2744 pos\_1904 pos\_1705 pos\_2054 pos\_730 pos\_499 pos\_1430 pos\_1709 pos\_101 pos\_1018 pos\_1087 pos\_507 pos\_2263 pos\_1355 pos\_57 pos\_1 pos\_722 pos\_83 pos\_577 pos\_1652 pos\_1019 pos\_2105 pos\_1007 pos\_2166 pos\_463 pos\_1980 pos\_1165 pos\_1619 pos\_813 pos\_2727 |
| Membrane transport | 02010 ABC transporters | 23 | pos\_1267 pos\_806 pos\_1048 pos\_2 pos\_862 pos\_2785 pos\_39 pos\_1697 pos\_492 pos\_1422 pos\_766 pos\_835 pos\_1044 pos\_4 pos\_1362 pos\_1025 pos\_650 pos\_2105 pos\_13 pos\_2612 pos\_212 pos\_2792 pos\_19 |
| Membrane transport | 02060 Phosphotransferase system (PTS) | 23 | pos\_1267 pos\_806 pos\_1048 pos\_2 pos\_862 pos\_2785 pos\_39 pos\_1697 pos\_492 pos\_1422 pos\_766 pos\_835 pos\_1044 pos\_4 pos\_1362 pos\_1025 pos\_650 pos\_2105 pos\_13 pos\_2612 pos\_212 pos\_2792 pos\_19 |
| Membrane transport | 03070 Bacterial secretion system | 23 | pos\_1267 pos\_806 pos\_1048 pos\_2 pos\_862 pos\_2785 pos\_39 pos\_1697 pos\_492 pos\_1422 pos\_766 pos\_835 pos\_1044 pos\_4 pos\_1362 pos\_1025 pos\_650 pos\_2105 pos\_13 pos\_2612 pos\_212 pos\_2792 pos\_19 |
| Metabolism of cofactors and vitamins | 00130 Ubiquinone and other terpenoid-quinone biosynthesis | 35 | pos\_1752 pos\_1286 pos\_913 pos\_1986 pos\_2601 pos\_388 pos\_2802 pos\_1048 pos\_1573 pos\_545 pos\_862 pos\_2466 pos\_1195 pos\_75 pos\_1260 pos\_478 pos\_614 pos\_2853 pos\_1370 pos\_2843 pos\_9 pos\_1844 pos\_1383 pos\_152 pos\_1740 pos\_220 pos\_2105 pos\_639 pos\_662 pos\_1022 pos\_1517 pos\_2218 pos\_754 pos\_911 pos\_272 |
| Metabolism of cofactors and vitamins | 00670 One carbon pool by folate | 19 | pos\_913 pos\_1986 pos\_2601 pos\_1573 pos\_545 pos\_862 pos\_1195 pos\_75 pos\_478 pos\_614 pos\_1370 pos\_2843 pos\_1740 pos\_2105 pos\_639 pos\_1022 pos\_1517 pos\_754 pos\_911 |
| Metabolism of cofactors and vitamins | 00730 Thiamine metabolism | 3 | pos\_2601 pos\_1740 pos\_2105 |
| Metabolism of cofactors and vitamins | 00740 Riboflavin metabolism | 4 | pos\_2601 pos\_1370 pos\_1740 pos\_2105 |
| Metabolism of cofactors and vitamins | 00750 Vitamin B6 metabolism | 5 | pos\_2105 pos\_1370 pos\_2601 pos\_911 pos\_1740 |
| Metabolism of cofactors and vitamins | 00760 Nicotinate and nicotinamide metabolism | 10 | pos\_1573 pos\_545 pos\_1195 pos\_2105 pos\_1370 pos\_1022 pos\_2601 pos\_911 pos\_1740 pos\_614 |
| Metabolism of cofactors and vitamins | 00770 Pantothenate and CoA biosynthesis | 12 | pos\_1573 pos\_545 pos\_1195 pos\_2105 pos\_913 pos\_1370 pos\_75 pos\_1022 pos\_2601 pos\_911 pos\_1740 pos\_614 |
| Metabolism of cofactors and vitamins | 00780 Biotin metabolism | 16 | pos\_1573 pos\_545 pos\_862 pos\_1195 pos\_2105 pos\_913 pos\_1370 pos\_75 pos\_1022 pos\_1517 pos\_2843 pos\_2601 pos\_754 pos\_911 pos\_1740 pos\_614 |
| Metabolism of cofactors and vitamins | 00785 Lipoic acid metabolism | 17 | pos\_1573 pos\_545 pos\_862 pos\_1195 pos\_2105 pos\_913 pos\_1370 pos\_75 pos\_1022 pos\_1517 pos\_2843 pos\_2601 pos\_754 pos\_478 pos\_911 pos\_1740 pos\_614 |
| Metabolism of cofactors and vitamins | 00790 Folate biosynthesis | 19 | pos\_913 pos\_1986 pos\_2601 pos\_1573 pos\_545 pos\_862 pos\_1195 pos\_75 pos\_478 pos\_614 pos\_1370 pos\_2843 pos\_1740 pos\_2105 pos\_639 pos\_1022 pos\_1517 pos\_754 pos\_911 |
| Metabolism of cofactors and vitamins | 00830 Retinol metabolism | 23 | pos\_913 pos\_1986 pos\_2601 pos\_1573 pos\_545 pos\_862 pos\_1195 pos\_75 pos\_1260 pos\_478 pos\_614 pos\_1370 pos\_2843 pos\_9 pos\_1740 pos\_220 pos\_2105 pos\_639 pos\_662 pos\_1022 pos\_1517 pos\_754 pos\_911 |
| Metabolism of cofactors and vitamins | 00860 Porphyrin and chlorophyll metabolism | 27 | pos\_1286 pos\_913 pos\_1986 pos\_2601 pos\_1048 pos\_1573 pos\_545 pos\_862 pos\_1195 pos\_75 pos\_1260 pos\_478 pos\_614 pos\_1370 pos\_2843 pos\_9 pos\_152 pos\_1740 pos\_220 pos\_2105 pos\_639 pos\_662 pos\_1022 pos\_1517 pos\_2218 pos\_754 pos\_911 |
| Metabolism of other amino acids | 00410 beta-Alanine metabolism | 3 | pos\_913 pos\_975 pos\_1267 |
| Metabolism of other amino acids | 00430 Taurine and hypotaurine metabolism | 5 | pos\_1267 pos\_913 pos\_975 pos\_1675 pos\_1745 |
| Metabolism of other amino acids | 00440 Phosphonate and phosphinate metabolism | 7 | pos\_2105 pos\_1267 pos\_913 pos\_975 pos\_431 pos\_1675 pos\_1745 |
| Metabolism of other amino acids | 00450 Selenocompound metabolism | 7 | pos\_2105 pos\_1267 pos\_913 pos\_975 pos\_431 pos\_1675 pos\_1745 |
| Metabolism of other amino acids | 00460 Cyanoamino acid metabolism | 7 | pos\_2105 pos\_1267 pos\_913 pos\_975 pos\_431 pos\_1675 pos\_1745 |
| Metabolism of other amino acids | 00471 D-Glutamine and D-glutamate metabolism | 7 | pos\_2105 pos\_1267 pos\_913 pos\_975 pos\_431 pos\_1675 pos\_1745 |
| Metabolism of other amino acids | 00472 D-Arginine and D-ornithine metabolism | 9 | pos\_766 pos\_2105 pos\_1267 pos\_1027 pos\_913 pos\_975 pos\_431 pos\_1675 pos\_1745 |
| Metabolism of other amino acids | 00473 D-Alanine metabolism | 9 | pos\_766 pos\_2105 pos\_1267 pos\_1027 pos\_913 pos\_975 pos\_431 pos\_1675 pos\_1745 |
| Metabolism of other amino acids | 00480 Glutathione metabolism | 11 | pos\_545 pos\_766 pos\_2105 pos\_1267 pos\_1027 pos\_913 pos\_975 pos\_431 pos\_1675 pos\_759 pos\_1745 |
| Metabolism of terpenoids and polyketides | 00253 Tetracycline biosynthesis | 4 | pos\_743 pos\_57 pos\_1783 pos\_1054 |
| Metabolism of terpenoids and polyketides | 00281 Geraniol degradation | 4 | pos\_743 pos\_57 pos\_1783 pos\_1054 |
| Metabolism of terpenoids and polyketides | 00522 Biosynthesis of 12-, 14- and 16-membered macrolides | 4 | pos\_743 pos\_57 pos\_1783 pos\_1054 |
| Metabolism of terpenoids and polyketides | 00523 Polyketide sugar unit biosynthesis | 4 | pos\_743 pos\_57 pos\_1783 pos\_1054 |
| Metabolism of terpenoids and polyketides | 00900 Terpenoid backbone biosynthesis | 2 | pos\_1783 pos\_1054 |
| Metabolism of terpenoids and polyketides | 00902 Monoterpenoid biosynthesis | 2 | pos\_1783 pos\_1054 |
| Metabolism of terpenoids and polyketides | 00903 Limonene and pinene degradation | 4 | pos\_743 pos\_57 pos\_1783 pos\_1054 |
| Metabolism of terpenoids and polyketides | 00904 Diterpenoid biosynthesis | 2 | pos\_1783 pos\_1054 |
| Metabolism of terpenoids and polyketides | 00905 Brassinosteroid biosynthesis | 2 | pos\_1783 pos\_1054 |
| Metabolism of terpenoids and polyketides | 00906 Carotenoid biosynthesis | 2 | pos\_1783 pos\_1054 |
| Metabolism of terpenoids and polyketides | 00908 Zeatin biosynthesis | 4 | pos\_743 pos\_57 pos\_1783 pos\_1054 |
| Metabolism of terpenoids and polyketides | 00909 Sesquiterpenoid and triterpenoid biosynthesis | 2 | pos\_1783 pos\_1054 |
| Metabolism of terpenoids and polyketides | 00981 Insect hormone biosynthesis | 4 | pos\_743 pos\_57 pos\_1783 pos\_1054 |
| Metabolism of terpenoids and polyketides | 01051 Biosynthesis of ansamycins | 4 | pos\_743 pos\_57 pos\_1783 pos\_1054 |
| Metabolism of terpenoids and polyketides | 01052 Type I polyketide structures | 4 | pos\_743 pos\_57 pos\_1783 pos\_1054 |
| Metabolism of terpenoids and polyketides | 01053 Biosynthesis of siderophore group nonribosomal peptides | 4 | pos\_743 pos\_57 pos\_1783 pos\_1054 |
| Metabolism of terpenoids and polyketides | 01054 Nonribosomal peptide structures | 4 | pos\_743 pos\_57 pos\_1783 pos\_1054 |
| Metabolism of terpenoids and polyketides | 01055 Biosynthesis of vancomycin group antibiotics | 4 | pos\_743 pos\_57 pos\_1783 pos\_1054 |
| Metabolism of terpenoids and polyketides | 01056 Biosynthesis of type II polyketide backbone | 4 | pos\_743 pos\_57 pos\_1783 pos\_1054 |
| Metabolism of terpenoids and polyketides | 01057 Biosynthesis of type II polyketide products | 4 | pos\_743 pos\_57 pos\_1783 pos\_1054 |
| Metabolism of terpenoids and polyketides | 01059 Biosynthesis of enediyne antibiotics | 4 | pos\_743 pos\_57 pos\_1783 pos\_1054 |
| Nervous system | 04720 Long-term potentiation | 9 | pos\_577 pos\_723 pos\_2166 pos\_1442 pos\_39 pos\_1982 pos\_879 pos\_607 pos\_1714 |
| Nervous system | 04721 Synaptic vesicle cycle | 18 | pos\_577 pos\_723 pos\_2166 pos\_1007 pos\_2105 pos\_1442 pos\_39 pos\_1982 pos\_1833 pos\_331 pos\_1430 pos\_2730 pos\_884 pos\_1018 pos\_1806 pos\_879 pos\_607 pos\_1714 |
| Nervous system | 04722 Neurotrophin signaling pathway | 18 | pos\_577 pos\_723 pos\_2166 pos\_1007 pos\_2105 pos\_1442 pos\_39 pos\_1982 pos\_1833 pos\_331 pos\_1430 pos\_2730 pos\_884 pos\_1018 pos\_1806 pos\_879 pos\_607 pos\_1714 |
| Nervous system | 04723 Retrograde endocannabinoid signaling | 16 | pos\_577 pos\_723 pos\_2166 pos\_1007 pos\_1442 pos\_39 pos\_1982 pos\_1833 pos\_331 pos\_1430 pos\_884 pos\_1018 pos\_1806 pos\_879 pos\_607 pos\_1714 |
| Nervous system | 04724 Glutamatergic synapse | 1 | pos\_1442 |
| Nervous system | 04725 Cholinergic synapse | 3 | pos\_1442 pos\_879 pos\_39 |
| Nervous system | 04726 Serotonergic synapse | 9 | pos\_577 pos\_723 pos\_2166 pos\_1442 pos\_39 pos\_1982 pos\_879 pos\_607 pos\_1714 |
| Nervous system | 04727 GABAergic synapse | 1 | pos\_1442 |
| Nervous system | 04728 Dopaminergic synapse | 3 | pos\_1442 pos\_879 pos\_39 |
| Nervous system | 04730 Long-term depression | 9 | pos\_577 pos\_723 pos\_2166 pos\_1442 pos\_39 pos\_1982 pos\_879 pos\_607 pos\_1714 |
| Neurodegenerative disease | 05012 Parkinson disease | 2 | pos\_1442 pos\_1025 |
| Neurodegenerative disease | 05014 Amyotrophic lateral sclerosis | 3 | pos\_1442 pos\_766 pos\_1025 |
| Neurodegenerative disease | 05016 Huntington disease | 3 | pos\_1442 pos\_766 pos\_1025 |
| Neurodegenerative disease | 05017 Spinocerebellar ataxia | 3 | pos\_1442 pos\_766 pos\_1025 |
| Neurodegenerative disease | 05020 Prion disease | 4 | pos\_1442 pos\_766 pos\_1025 pos\_1148 |
| Neurodegenerative disease | 05022 Pathways of neurodegeneration - multiple diseases | 4 | pos\_1442 pos\_766 pos\_1025 pos\_1148 |
| Nucleotide metabolism | 00230 Purine metabolism | 13 | pos\_2717 pos\_12 pos\_2105 pos\_2328 pos\_15 pos\_13 pos\_1860 pos\_1025 pos\_92 pos\_1810 pos\_564 pos\_61 pos\_602 |
| Nucleotide metabolism | 00240 Pyrimidine metabolism | 16 | pos\_2717 pos\_2406 pos\_12 pos\_2105 pos\_2328 pos\_15 pos\_13 pos\_1860 pos\_1025 pos\_92 pos\_1810 pos\_564 pos\_61 pos\_212 pos\_2792 pos\_602 |
| Sensory system | 04740 Olfactory transduction | 4 | pos\_1442 pos\_1355 pos\_220 pos\_1714 |
| Sensory system | 04742 Taste transduction | 6 | pos\_1442 pos\_5 pos\_879 pos\_1355 pos\_220 pos\_1714 |
| Sensory system | 04744 Phototransduction | 1 | pos\_220 |
| Sensory system | 04745 Phototransduction - fly | 4 | pos\_1442 pos\_1355 pos\_220 pos\_1714 |
| Sensory system | 04750 Inflammatory mediator regulation of TRP channels | 10 | pos\_1442 pos\_5 pos\_1982 pos\_884 pos\_362 pos\_2730 pos\_879 pos\_1355 pos\_220 pos\_1714 |
| Signal transduction | 04010 MAPK signaling pathway | 1 | pos\_1442 |
| Signal transduction | 04011 MAPK signaling pathway - yeast | 1 | pos\_1442 |
| Signal transduction | 04012 ErbB signaling pathway | 1 | pos\_1442 |
| Signal transduction | 04013 MAPK signaling pathway - fly | 1 | pos\_1442 |
| Signal transduction | 04014 Ras signaling pathway | 1 | pos\_1442 |
| Signal transduction | 04015 Rap1 signaling pathway | 1 | pos\_1442 |
| Signal transduction | 04016 MAPK signaling pathway - plant | 1 | pos\_1442 |
| Signal transduction | 04020 Calcium signaling pathway | 2 | pos\_1442 pos\_2601 |
| Signal transduction | 04022 cGMP-PKG signaling pathway | 7 | pos\_1442 pos\_1982 pos\_2601 pos\_1709 pos\_1025 pos\_1 pos\_879 |
| Signal transduction | 04024 cAMP signaling pathway | 7 | pos\_1442 pos\_1982 pos\_2601 pos\_1709 pos\_1025 pos\_1 pos\_879 |
| Signal transduction | 04064 NF-kappa B signaling pathway | 1 | pos\_1442 |
| Signal transduction | 04066 HIF-1 signaling pathway | 1 | pos\_1442 |
| Signal transduction | 04068 FoxO signaling pathway | 1 | pos\_1442 |
| Signal transduction | 04070 Phosphatidylinositol signaling system | 2 | pos\_1442 pos\_2601 |
| Signal transduction | 04071 Sphingolipid signaling pathway | 5 | pos\_1442 pos\_2601 pos\_1709 pos\_1025 pos\_1 |
| Signal transduction | 04072 Phospholipase D signaling pathway | 2 | pos\_1442 pos\_2601 |
| Signal transduction | 04075 Plant hormone signal transduction | 9 | pos\_766 pos\_1442 pos\_1982 pos\_1044 pos\_2601 pos\_1709 pos\_1025 pos\_1 pos\_879 |
| Signal transduction | 04150 mTOR signaling pathway | 9 | pos\_766 pos\_1442 pos\_1982 pos\_1044 pos\_2601 pos\_1709 pos\_1025 pos\_1 pos\_879 |
| Signal transduction | 04151 PI3K-Akt signaling pathway | 7 | pos\_1442 pos\_1982 pos\_2601 pos\_1709 pos\_1025 pos\_1 pos\_879 |
| Signal transduction | 04152 AMPK signaling pathway | 7 | pos\_1442 pos\_1982 pos\_2601 pos\_1709 pos\_1025 pos\_1 pos\_879 |
| Signal transduction | 04310 Wnt signaling pathway | 1 | pos\_1442 |
| Signal transduction | 04330 Notch signaling pathway | 1 | pos\_1442 |
| Signal transduction | 04340 Hedgehog signaling pathway | 1 | pos\_1442 |
| Signal transduction | 04341 Hedgehog signaling pathway - fly | 1 | pos\_1442 |
| Signal transduction | 04350 TGF-beta signaling pathway | 1 | pos\_1442 |
| Signal transduction | 04370 VEGF signaling pathway | 1 | pos\_1442 |
| Signal transduction | 04371 Apelin signaling pathway | 1 | pos\_1442 |
| Signal transduction | 04390 Hippo signaling pathway | 1 | pos\_1442 |
| Signal transduction | 04391 Hippo signaling pathway - fly | 1 | pos\_1442 |
| Signal transduction | 04392 Hippo signaling pathway - multiple species | 1 | pos\_1442 |
| Signal transduction | 04630 JAK-STAT signaling pathway | 1 | pos\_1442 |
| Signal transduction | 04668 TNF signaling pathway | 1 | pos\_1442 |
| Signaling molecules and interaction | 04060 Cytokine-cytokine receptor interaction | 7 | pos\_2105 pos\_819 pos\_1982 pos\_884 pos\_1025 pos\_2730 pos\_879 |
| Signaling molecules and interaction | 04061 Viral protein interaction with cytokine and cytokine receptor | 7 | pos\_2105 pos\_819 pos\_1982 pos\_884 pos\_1025 pos\_2730 pos\_879 |
| Signaling molecules and interaction | 04080 Neuroactive ligand-receptor interaction | 7 | pos\_2105 pos\_819 pos\_1982 pos\_884 pos\_1025 pos\_2730 pos\_879 |
| Signaling molecules and interaction | 04512 ECM-receptor interaction | 7 | pos\_2105 pos\_819 pos\_1982 pos\_884 pos\_1025 pos\_2730 pos\_879 |
| Signaling molecules and interaction | 04514 Cell adhesion molecules | 7 | pos\_2105 pos\_819 pos\_1982 pos\_884 pos\_1025 pos\_2730 pos\_879 |
| Substance dependence | 05032 Morphine addiction | 1 | pos\_1025 |
| Substance dependence | 05033 Nicotine addiction | 2 | pos\_879 pos\_1025 |
| Substance dependence | 05034 Alcoholism | 2 | pos\_879 pos\_1025 |
| Translation | 00970 Aminoacyl-tRNA biosynthesis | 8 | pos\_862 pos\_766 pos\_2105 pos\_1267 pos\_1044 pos\_4 pos\_671 pos\_1048 |
| Translation | 03008 Ribosome biogenesis in eukaryotes | 8 | pos\_862 pos\_766 pos\_2105 pos\_1267 pos\_1044 pos\_4 pos\_671 pos\_1048 |
| Translation | 03013 RNA transport | 8 | pos\_862 pos\_766 pos\_2105 pos\_1267 pos\_1044 pos\_4 pos\_671 pos\_1048 |
| Translation | 03015 mRNA surveillance pathway | 8 | pos\_862 pos\_766 pos\_2105 pos\_1267 pos\_1044 pos\_4 pos\_671 pos\_1048 |
| Transport and catabolism | 04136 Autophagy - other | 2 | pos\_1007 pos\_331 |
| Transport and catabolism | 04137 Mitophagy - animal | 2 | pos\_1007 pos\_331 |
| Transport and catabolism | 04138 Autophagy - yeast | 2 | pos\_1007 pos\_331 |
| Transport and catabolism | 04139 Mitophagy - yeast | 2 | pos\_1007 pos\_331 |
| Transport and catabolism | 04140 Autophagy - animal | 2 | pos\_1007 pos\_331 |
| Xenobiotics biodegradation and metabolism | 00980 Metabolism of xenobiotics by cytochrome P450 | 9 | pos\_2008 pos\_1195 pos\_2634 pos\_2463 pos\_2330 pos\_2509 pos\_181 pos\_395 pos\_326 |
| Xenobiotics biodegradation and metabolism | 00982 Drug metabolism - cytochrome P450 | 13 | pos\_2008 pos\_2191 pos\_1195 pos\_2634 pos\_2465 pos\_2463 pos\_2283 pos\_2330 pos\_2567 pos\_2509 pos\_181 pos\_395 pos\_326 |
| Xenobiotics biodegradation and metabolism | 00983 Drug metabolism - other enzymes | 16 | pos\_2008 pos\_545 pos\_2191 pos\_1195 pos\_2634 pos\_2465 pos\_2463 pos\_2283 pos\_2330 pos\_2567 pos\_1539 pos\_2601 pos\_2509 pos\_181 pos\_395 pos\_326 |
